# Arterial spin labeled MRI in prodromal Alzheimer's disease: A multi-site study^☆^

**DOI:** 10.1016/j.nicl.2013.04.014

**Published:** 2013-04-30

**Authors:** Ze Wang, Sandhitsu R. Das, Sharon X. Xie, Steven E. Arnold, John A. Detre, David A. Wolk, Alzheimer's Disease Neuroimaging Initiative

**Affiliations:** a Department of Neurology, University of Pennsylvania, Philadelphia, PA, USA; b Department of Radiology, University of Pennsylvania, Philadelphia, PA, USA; c Department of Psychiatry, University of Pennsylvania, Philadelphia, PA, USA; d Department of Biostatistics and Epidemiology, University of Pennsylvania, Philadelphia, PA, USA; e Center for Functional Neuroimaging, University of Pennsylvania, Philadelphia, PA, USA; f Penn Memory Center, University of Pennsylvania, Philadelphia, PA, USA

## Abstract

We examined differences in cerebral blood flow (CBF) measured by Arterial Spin Labeled perfusion MRI (ASL MRI) across the continuum from cognitively normal (CN) older adults to mild Alzheimer's Disease (AD) using data from the multi-site Alzheimer's Disease Neuroimaging Initiative (ADNI). Measures of CBF, in a predetermined set of regions (meta-ROI), and hippocampal volume were compared between CN (n = 47), patients with early and late Mild Cognitive Impairment [EMCI (n = 32), LMCI (n = 35)], and AD (n = 15). Associations between these measures and disease severity, assessed by Clinical Dementia Rating scale sum of boxes (CDR SB), were also assessed. Mean meta-ROI CBF was associated with group status and significant hypoperfusion was observed in LMCI and AD relative to CN. Hippocampal volume was associated with group status, but only AD patients had significantly smaller volumes than the CN. When examining the relationship between these measures and disease severity, both were significantly associated with CDR SB and appeared to provide independent prediction of status. In light of the tight link between CBF and metabolism, ASL MRI represents a promising functional biomarker for early diagnosis and disease tracking in AD and this study is the first to demonstrate the feasibility in a multi-site context in this population. Combining functional and structural measures, which can be acquired in the same scanning session, appears to provide additional information about disease severity relative to either measure alone.

## Highlights

• Arterial Spin Labeled (ASL) MRI is a promising AD biomarker. No prior multi-site study of this modality in AD.
• We measured cerebral blood flow (CBF) in a FDG PET defined region.
• Reduced CBF was associated with MCI and AD and correlated with disease severity.
• Hippocampal volume and ASL provide complementary information.
• ASL MRI can be applied to this population in a multi-site context.

## 1 Introduction

Alzheimer's disease (AD) is associated with alterations in regional cerebral blood flow (CBF) that overlap with well-described abnormalities in cerebral metabolism measured by ^18^F-fluorodeoxyglucose positron emission tomography (FDG PET) (Chen et al., 2011; Ishii et al., 1997; Jagust et al., 2001; Johnson et al., 1998), consistent with the generally tight link between CBF and brain metabolism (Baron et al., 1982; Fox and Raichle, 1986). While CBF has traditionally been measured in the clinical setting using radioactive tracers and nuclear medicine techniques, arterial spin labeled (ASL) perfusion MRI offers a non-invasive approach to CBF measurement (Detre et al., 1992) that can be obtained in conjunction with structural MRI scanning. ASL MRI utilizes magnetically labeled blood water as an endogenous tracer for quantification of brain perfusion, and does not require injections or exposure to ionizing radiation (Detre et al., 2009, 2012).

Over the last several years, ASL MRI has been applied to prodromal and clinical AD patient cohorts, displaying sensitivity to early disease stages (Alexopoulos et al., 2012; Alsop et al., 2000, 2010; Chao et al., 2009, 2010; Dai et al., 2009; Hu et al., 2010; Johnson et al., 2005; Wolk and Detre, 2012; Xu et al., 2007; Yoshiura et al., 2009). When directly compared, there appears to be a high degree of concordance between ASL MRI and both FDG PET (Chen et al., 2011; Musiek et al., 2012) and O15 PET (Xu et al., 2010). FDG PET is considered a particularly useful ‘neurodegenerative’ biomarker sensitive to the early functional downstream effects of AD-related pathology (Jack et al., 2010) and complementary to measures that reflect the molecular pathology of AD, such as amyloid imaging. FDG PET has been incorporated into recently proposed criteria of prodromal and clinical AD (Albert et al., 2011; McKhann et al., 2011) and provides a potential outcome measure for evidence of treatment effects in clinical trials of these populations (K. Chen et al., 2010; Jack et al., 2010). The availability of an MRI-based neurodegenerative biomarker analogous to FDG PET would be desirable due to greater accessibility and lower cost of MRI relative to PET, while the lack of ionizing radiation exposure is a safety benefit, particularly for longitudinal studies. ASL MRI can also be obtained concomitantly with structural MRI, which also provides a valuable biomarker of neurodegeneration (Jack et al., 2010).

All prior works with ASL MRI in AD populations have been performed at a single site, often with idiosyncratic research-based sequences, and few studies in even healthy cohorts have involved multiple sites (Binnewijzend et al., 2012; Okonkwo et al., 2013; Petersen et al., 2010). However, for ASL MRI to be realized as a valuable tool for clinical research, it must be validated in a multi-site setting. The Alzheimer's Disease Neuroimaging Initiative 2 (ADNI 2) includes a sub-study of ASL MRI for participants scanned on the Siemens 3T MRI platform (~ 1/3 of enrolled subjects) using a commercial ASL sequence [pulsed ASL; PICORE-Q2TIPS (Luh et al., 1999; Wong et al., 1997)]. This multi-site study allows for the assessment of ASL MRI sensitivity to disease severity across the spectrum from cognitively normal adults, early and late mild cognitive impairment (EMCI, LMCI), and mild AD. In the present study, we examined differences in CBF across these groups using a composite region of interest (ROI) previously found to be sensitive to AD-related CBF changes (Chen et al., 2011) and originally developed for FDG PET data (Landau et al., 2010, 2011). To provide context, we compared this measure to that of hippocampal volume, a commonly used AD biomarker in this population. As hippocampal volume can be and is obtained during the same scanning session as the ASL sequence, we also explored whether these measures together can provide complementary information that can be used in tandem to enhance description of disease status.

## 2 Materials and methods

### 2.1 Participants

Data used in the preparation of this article were obtained from the Alzheimer's Disease Neuroimaging Initiative (ADNI) database (adni.loni.ucla.edu). The ADNI was launched in 2003 by the National Institute on Aging (NIA), the National Institute of Biomedical Imaging and Bioengineering (NIBIB), the Food and Drug Administration (FDA), private pharmaceutical companies and non-profit organizations, as a $60 million, 5-year public private partnership. The primary goal of ADNI has been to test whether serial magnetic resonance imaging (MRI), positron emission tomography (PET), other biological markers, and clinical and neuropsychological assessment can be combined to measure the progression of mild cognitive impairment (MCI) and early Alzheimer's disease (AD). Determination of sensitive and specific markers of very early AD progression is intended to aid researchers and clinicians to develop new treatments and monitor their effectiveness, as well as lessen the time and cost of clinical trials.

The Principal Investigator of this initiative is Michael W. Weiner, MD, VA Medical Center and University of California — San Francisco. ADNI is the result of efforts of many co investigators from a broad range of academic institutions and private corporations, and subjects have been recruited from over 50 sites across the U.S. and Canada. The initial goal of ADNI was to recruit 800 subjects but ADNI has been followed by ADNI-GO and ADNI-2. To date these three protocols have recruited over 1500 adults, ages 55 to 90, to participate in the research, consisting of cognitively normal older individuals, people with early or late MCI (EMCI or LMCI), and people with early AD. The follow up duration of each group is specified in the protocols for ADNI-1, ADNI-2 and ADNI-GO. Subjects originally recruited for ADNI-1 and ADNI-GO had the option to be followed in ADNI-2. For up-to-date information, see www.adni-info.org.

Data for the current manuscript were limited to baseline scans from ADNI 2 participants in the ASL MRI substudy as of May, 2012. This cohort included 47 cognitively normal adults [CN; age: 73.1 ± 7.0 yrs (mean ± standard deviation), Mini-Mental Status Examination (MMSE): 28.9 ± 1.3, 20 males, 27 females], 15 Alzheimer's disease (AD) patients (age: 75.6 ± 8.8 yrs, MMSE: 22.4 ± 1.6, 11 males, 4 females), 32 EMCI patients (age: 68.9 ± 7.1, MMSE: 28.6 ± 1.3, 18 males, 14 females), and 35 LMCI patients (age: 72.2 ± 7.4, MMSE: 27.5 ± 2.0, 18 males, 17 females). Full inclusion and exclusion criteria for ADNI are described at www.adni-info.org. In brief, patients with MCI were classified essentially in the manner described by Petersen (2004), but were then further divided into an “early” and “late” group based on performance on the Wechsler Memory Scale–Revised Logical Memory II (WMS-LM). The EMCI group was defined based on scores between the cutoff of normal and that of the LMCI group. Nine individuals (4 CN, 1 EMCI, 4 LMCI) were excluded due to poor ASL MRI data quality (see below) and one CN adult did not have hippocampal volume measured. Detailed site information and the number of subjects in each sub-group whose ASL MRI were included or excluded are listed in Supplementary Table 1.

### 2.2 Image acquisition

Both high-resolution structural MRI data and resting ASL data were downloaded. The structural images were acquired using a 3D MPRAGE T1-weighted sequence with the following parameters: TR/TE/TI = 2300/2.98/900 ms, 176 sagittal slices, within plane FOV = 256 × 240 mm^2^, voxel size = 1.1 × 1.1 × 1.2 mm^3^, flip angle = 9°, bandwidth = 240 Hz/pix. ASL data were acquired using the Siemens product PICORE sequence (Wong et al., 1997), which is a pulsed ASL (PASL) sequence using the Q2TIPs (Luh et al., 1999) technique for defining the spin bolus. The acquisition parameters were: TR/TE = 3400/12 ms, TI1/TI2 = 700/1900 ms, FOV = 256 mm, 24 sequential 4 mm thick slices with a 25% gap between the adjacent slices, partial Fourier factor = 6/8, bandwidth = 2368 Hz/pix, and imaging matrix = 64 × 64. The first volume of the 105 ASL acquisitions was used as the M0 image.

### 2.3 Image processing and analysis

Image processing used SPM8 (http://www.fil.ion.ucl.ac.uk/spm), FSL (http://www.fmrib.ox.ac.uk/fsl/), and an in-house package developed at University of Pennsylvania for automatic structural segmentation. The mean control image was registered to the high resolution structural image. Structural images were segmented into gray matter (GM), white matter (WM), and CSF using the segmentation tool provided in SPM8, which were projected into the ASL image space based on the registration correspondence between the mean ASL control image and the structural image. The projected WM and CSF segments were then used to extract the mean WM and CSF signal from the ASL image series.

The Diffeomorphic Anatomical Registration Through Exponential Lie Algebra (DARTEL) routine (Ashburner, 2007) implemented in SPM8 was used to generate a local template for all subjects based on their segmented gray matter and white matter probability maps and the local template was registered into the MNI standard space using a linear affine transformation. With these two transforms, each individual subject's brain was mapped into the MNI space. The same combined transform was also used to map each subject's mean CBF map into the MNI space.

ASL images were preprocessed using the pipeline implemented in ASLtbx (Wang et al., 2008) (see a scheme of the pipeline in Supplementary Fig. 1) The first step was motion correction (MoCo) (Wang, 2012) and denoising. Denoising included spatial smoothing with an isotropic Gaussian at full-width-at-half-maximum (FWHM) of 4 mm^3^, temporal filtering using a high-pass Butterworth filter (cutoff frequency = 0.01 Hz) and temporal nuisance cleaning. The skull was stripped using the FSL BET tool to generate a brain mask. This mask was then used to extract the global mean signal timecourse, excluding extracranial voxels. Temporal nuisances including head motion time courses (3 translations and 3 rotations), the global signal timecourse, the WM mean signal timecourse, and the CSF mean signal timecourse were regressed out from ASL image series at each voxel (Wang, 2012). The next step was pair-wise subtraction and CBF quantification using the one-compartment model (Buxton et al., 1998) implemented in ASLtbx. The detailed model parameters can be found be in Wang et al. (2008) and Cavusoglu et al. (2009).

The resulting CBF time series (52 images) was then cleaned using an adaptive procedure. Initial outliers were identified using the head motion time courses and the whole brain CBF time series as described above (Wang et al., 2008). For adaptive cleaning, the Pearson's correlation between the gray matter voxels of each ASL CBF time point and the mean of the remaining CBF time series was calculated. Time points with a correlation coefficient (CC) smaller than 0.15 (p < 1e− 6; the effective degrees of freedom for calculating CC was > 1000 for all subjects) or out of the range of mean ± 2 standard deviation of all image CCs were identified as new outliers and were excluded. The same iteration was repeated until no new outliers were identified. For most of the subjects, the procedure converged with one iteration, only 3 subjects took 2 iterations to converge. The remaining time points were averaged to generate the final CBF image. A detailed implementation procedure is illustrated in Supplementary Fig. 2.

Partial volume effect (PVE) correction was performed to correct CBF at each voxel in the gray matter using a previously described approach (Du et al., 2006). The PVE corrected CBF map was then registered into the structural image space using the same registration transform from the mean ASL control image to the structural image described above. Mean GM CBF within the PET data-derived meta-region-of-interest (meta-ROI) previously reported by Landau et al. (2011) was extracted for all subjects. This meta-ROI consists of spheres in precuneus, bilateral parietal cortex, and bilateral temporal cortex. A spherical ROI within the visual cortex was used as a control for the meta-ROI and mean CBF was extracted from this control ROI as well. The location of the meta-ROI and visual cortex ROI on the MNI template can be seen in Supplementary Fig. 3. Average hippocampal volumes (left and right) were derived from T1-weighted structural data (Wang et al., 2011) and divided by ICV (GM + WM + CSF) for normalization.

### 2.4 Statistical analysis

Group differences in demographic data were determined by *χ*^2^ (for frequencies) and 2-sample t-tests. Separate linear regression analyses were performed with the meta-ROI or hippocampal volume as the outcome variable and group (4 levels; defined by 3 dummy variables) as the predictor variable. Age was included as a covariate. Planned pair-wise comparisons of each group with the CN group were also performed. Pearson correlation of the meta-ROI and hippocampal volume with measures of disease severity [i.e. Clinical Dementia Rating scale sum of boxes (CDR SB; (Morris, 1993)) and the MMSE; (Folstein et al., 1975)] were calculated. Finally, to determine the degree to which these measures independently predicted disease severity, a step-wise regression model was developed in which age and education were entered as the first step and meta-ROI and hippocampal volume were entered as the second in a step-wise manner; the default value of p < 0.05 to enter an independent variable into the model was employed. Statistical analyses were performed using SPSS 20.0 (Chicago, IL) and SAS version 9.32 (SAS Institute Inc., Cary, North Carolina). All statistical tests were two-sided. Statistical significance was set at the p < .05 level unless otherwise noted.

## 3 Results

### 3.1 Demographic and psychometric data

Demographic and psychometric data of the study population included in the ASL MRI analysis are provided in Table 1. EMCI patients were younger than the other three groups [CN: t(73) = 2.6, p < 0.05; LMCI: t(59) = 1.9, p < 0.06; AD: t(44) = 2.8, p < 0.01]. Not surprisingly, MMSE was progressively lower in EMCI, LMCI, and AD patients, respectively, relative to CN adults, but the EMCI group did not significantly differ from the CN group [t(73) = 1.4, p > 0.1]. Indeed, the only cognitive measure that clearly differed between the EMCI patients and CN adults was the WMS-LM, which, in part, defined category membership, and the CDR SB. LMCI and AD patients displayed significant impairment across domains relative to the CN group, but for most measures LMCI was within 1 SD of the CN group.

### 3.2 Data quality and cleaning

**Fig. 1:**
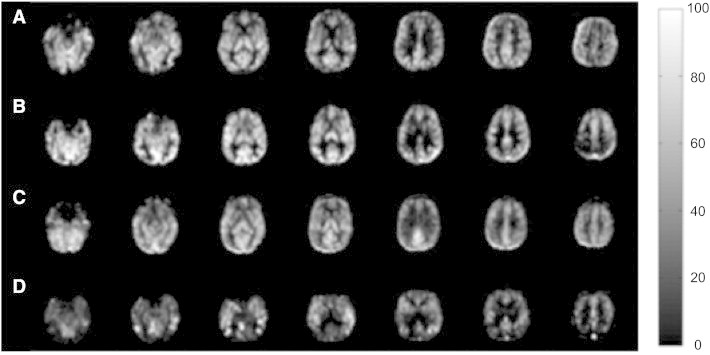
Four representative CBF images from (A) CN adult, (B) EMCI patient, (C) LMCI patient, and (D) an AD patient. The display window is from 0 to 100 ml/100 g/min.

Three subjects' ASL images did not cover a sufficient portion of the brain and were excluded from further analysis. After data cleaning, ASL MRI data from an additional 6 subjects were excluded due to extensive non-physiological negative CBF values in gray matter. These negative CBF regions showed a large standard deviation over time suggesting instability of spin labeling or hardware instabilities. A total of 9 subjects' data (~ 7%) were excluded from the ASL CBF analysis. The mean and standard deviation of the number of removed time points due to the adaptive data cleaning were 8.1 ± 2.7 (mean ± standard deviation), 9.0 ± 2.4, 9.8 ± 2.7, and 12.6 ± 6.7, for the HC, EMCI, LMCI, and AD sub-group, respectively. The number of removed time points increased with disease severity (from HC to AD) (one-way ANOVA, p < 0.001). AD patients had more bad time points removed than HC (2-sample t-test, two tailed, t(57) = 3.37, p < 0.001) and EMCI (2-sample t-test, two tailed, t(44) = 2.54, p < 0.02) and showed a trend difference compared to LMCI (2-sample t-test, two tailed, t(44) = 1.98, p = 0.054). LMCI had more bad time points removed than HC (2-sample t-test, two tailed, t(72) = 2.41 p < 0.02). Fig. 1 shows processed CBF images from 4 representative subjects (one from each subgroup). Note that the AD patient demonstrated the most visually obvious hypoperfusion in posterior brain region characteristic of this diagnosis. These scans came from 3 different scanner sites (LMCI and AD were from the same site) and, overall, there were no clear differences in ASL MRI quality across sites. Supplementary Table 1 provides details on included and excluded scans from each site.

### 3.3 Group comparisons of CBF in meta-ROI and hippocampal volume

**Fig. 2:**
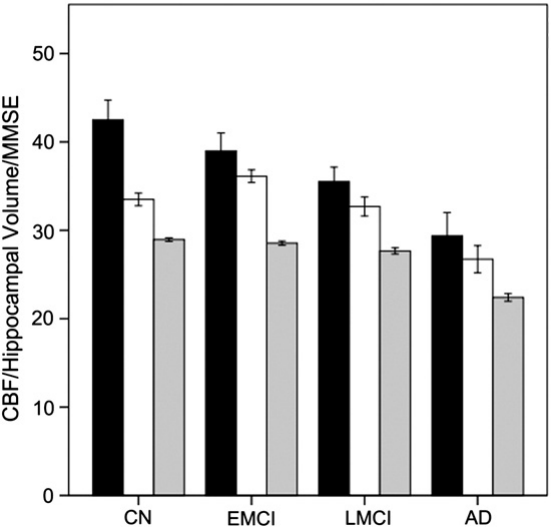
Cerebral blood flow (CBF), hippocampal volume, and MMSE for each of the four subgroups. Y-axis units are ml/100 g/min for CBF (black), mm^3^∗ 10,000/intracranial volume for hippocampal volume (white), and 30 points maximum for MMSE (gray). Error bars reflect ± 1 standard error of the mean.

Fig. 2 shows mean CBF of the meta-ROI and hippocampal volume across the 4 subgroups. CBF gradually decreased in the continuum from CN adults to patients with AD while only the LMCI and AD groups displayed smaller hippocampal volume relative to the CN group in absolute terms. To examine the relationship of CBF with group status, a regression model was developed with CBF as the outcome variable, group as the dependent variable, and age as a covariate. Group status was associated with CBF [F(3,115) = 5.1, p < 0.01] and the overall model was significant [F(4,115) = 3.85, p < 0.01]. When comparing the patient groups to the CN adults, we found that patients with AD [t(118) = 3.7, p < 0.001, Cohen's d = 1.09] and LMCI [t(118) = 2.9, p < 0.05, Cohen's d = 0.58] displayed significantly reduced CBF, but this difference did not reach significance in the EMCI group [t(118) = 1.1, p > 0.1, Cohen's d = 0.27]. Note that no group differences were found when using CBF of the visual cortex control ROI (p's > 0.15).

The same model with hippocampal volume as the outcome also revealed a significant effect of group [F(3,123) = 6.7, p < 0.001; model: F(4,123) = 9.5, p < 0.0001]. However, only the AD group significantly differed from CN adults [t(126) = 3.9, p < 0.001, Cohen's d = 1.30]. EMCI and LMCI patients did not display significantly smaller hippocampal volume compared to the CN group [EMCI: t(126) < 1.0, p > 0.1, Cohen's d = − 0.35; LMCI: t(126) = 1.5, p > 0.1, Cohen's d = 0.26].

### 3.4 Relationship of CBF and hippocampal volume to disease severity

**Fig. 3:**
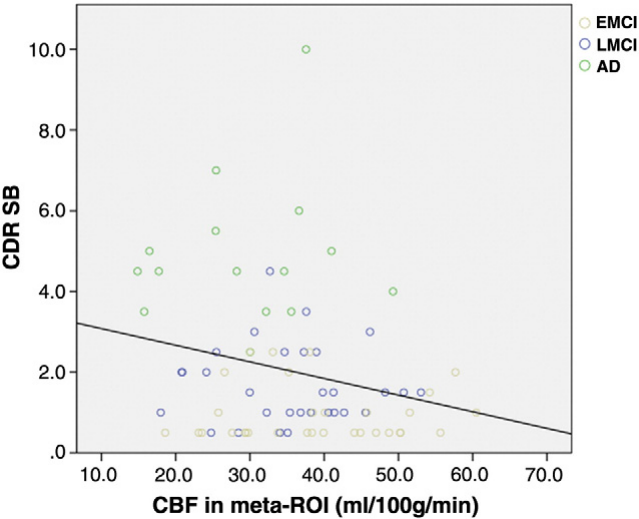
Meta-ROI CBF vs CDR SB score in the symptomatic patients.

When including the entire cohort with age and education as covariates, meta-ROI CBF was significantly correlated with CDR SB (r = − .32, p = 0.001). Similar correlations were observed using hippocampus volume as a predictive variable (hippocampus: r = − .38, p < 0.001) These correlations showed that lower CBF or smaller volume was associated with higher CDR SB (greater impairment). Similarly, if restricted to just symptomatic individuals (EMCI, LMCI, AD), both measures still correlated with disease severity (CBF: r = − .30, p < 0.05; hippocampus: r = − .42, p < 0.001). Fig. 3 is a plot of the meta-ROI CBF vs CDR SB score in the symptomatic patients. Analogous, though slightly weaker, correlations were found with MMSE.

As the primary cognitive deficit in this population is memory loss, the above correlations were repeated for the WMS-LM delayed recall. Across the entire cohort, CBF in the meta-ROI (r = .32, p < 0.001) and hippocampus (r = .32, p = 0.001) was correlated with this measure. Similarly, both measures were correlated with delayed recall when restricted to symptomatic patients (CBF: r = .31, p < 0.01; hippocampus: r = .46, p < 0.001). Perhaps not surprisingly, hippocampal volume appeared more strongly related to this measure given the role of this structure in memory function.

To assess whether CBF in the meta-ROI and hippocampal volume provided independent information regarding disease severity, a hierarchical regression model was developed in which age and education were entered in the first step and then CBF and hippocampal volume were included in a step-wise manner. Across the entire cohort, both measures (hippocampus: β = − .37, p < 0.001; CBF: β = − .29, p = 0.001) were included in the model with the highest explanatory power [F(4,118) = 9.2, p < 0.001]. Both measures were also included in the same model when restricted to symptomatic patients [hippocampus: β = − .44, p < 0.001; CBF: β = − .27, p < 0.01; F(4,75) = 8.2, p < 0.001]. Very similar results were found with MMSE as the dependent variable.

Finally, we performed the analogous regression using delayed recall on the WMS-LM test. Across the entire cohort, both measures (hippocampus: β = .31, p < 0.01; CBF: β = .30, p = 0.01) were included in the model with the highest explanatory power [F(4,118) = 8.7, p < 0.001]. Again, a similar result was found when restricted to the symptomatic patients (hippocampus: β = .46, p < 0.001; CBF: β = .28, p < 0.01; F(4,75) = 13.1, p < 0.001). Taken together, these findings suggest that CBF and hippocampal volume independently contribute to explanation of disease severity.

## 4 Discussion

Our results demonstrate the sensitivity of an ASL MRI-based biomarker to prodromal and early AD in a multisite context. While the pathoetiology of MCI is heterogeneous, ASL MRI meta-ROI data appears to track disease severity. Further, group discrimination by this measure was comparable to hippocampal volume measurement based on T1-weighted imaging, which is the most well studied neuroimaging biomarker in these populations. More importantly, ASL and structural MRI appeared to provide complementary information with regard to disease severity, which is an important finding given the ease of obtaining both of these image types in the same scanning session.

This finding adds to a growing literature suggesting that ASL MRI is a sensitive biomarker in the spectrum from MCI to early AD (Alexopoulos et al., 2012; Alsop et al., 2000, 2010; Chao et al., 2009, 2010; Chen et al., 2011; Dai et al., 2009; Hu et al., 2010; Johnson et al., 2005; Wolk and Detre, 2012; Xu et al., 2007; Yoshiura et al., 2009). The current study used a meta-ROI summary measure derived from the FDG PET literature to measure regional CBF (Landau et al., 2010, 2011). We previously demonstrated in a single-site study that mean CBF in this set of regions distinguished healthy controls from AD patients to a similar extent as FDG PET (Chen et al., 2011), and also significantly differentiated MCI from AD and controls (Zhang et al., 2012). The sensitivity of this same measure to MCI and AD patients in the ADNI cohort confirms these prior findings. Notably, a recent study that also included a subset of ADNI participants and applied the same meta-ROI to FDG PET data produced very similar results to the current findings (Landau et al., 2012), with little difference between CN and EMCI groups as found here, but more apparent hypometabolism in LMCI and AD patients.

To control for the regional specificity of the meta-ROI, we also examined CBF from an ROI within the visual cortex and didn't find CBF-disease associations, suggesting that the observed effects are unlikely to be a consequence of less specific, global CBF effects. In order to dissociate PVE from CBF, we performed PVE correction before the cross-sectional data analysis. We observed similar, but slightly stronger, cross-sectional differences without applying PVE correction (data not shown here).

Taken together, these data support the notion that ASL MRI provides largely overlapping findings with FDG PET (Foster et al., 2007; Jagust et al., 2009, 2010; Silverman et al., 2001). While it is not clear whether ASL MRI provides additional information in these contexts, it offers several advantages, including greater accessibility, lower expense, and lack of invasiveness or exposure to radiation. Moreover, the current findings demonstrate the potential utility of multi-modality data for disease monitoring, as CBF and hippocampal volume together provided the highest explanatory power for disease severity. ASL MRI can be obtained as a relatively short sequence (~ 6–8 min) within the context of a routine MRI, and may obviate the need for an additional FDG PET scan in many or all patients. This relative performance of ASL MRI and FDG PET can be further assessed in future analyses of the ADNI2 cohort.

The current study is limited to data acquired on the Siemens 3T platform and the number of subjects from each site in our data was too small to explicitly control for site effects. However we did visually inspect image quality and did not find perceivable CBF differences across different sites, as was illustrated in the sample images in Fig. 1. Although more formal approaches to testing site effects will be needed in future work, the current data is encouraging. These data, taken in combination with other single site studies using different platforms and different ASL acquisition techniques (Binnewijzend et al., 2012; Okonkwo et al., 2013),suggest that ASL MRI data can be successfully collected in a neurodegenerative population across multiple platforms or sites. It is also encouraging that significant effects could be observed using a commercial sequence (Luh et al., 1999; Wong et al., 1997) lacking advanced features. PASL is known to provide inferior SNR and test–retest reliability relative to pseudo-continuous ASL (Y. Chen et al., 2010; Wu et al., 2009); The pseudo-continuous ASL (PCASL) scheme inverts inflowing blood water over 1–2 s to achieve a higher spin labeling efficiency and therefore higher SNR for CBF estimation than PASL. And despite the absence of background suppression (Ye et al., 2000), over 90% of the acquired data was useable with implementation of advanced signal processing approaches. This suggests that future studies employing more advanced ASL methodologies should provide even greater sensitivity for detecting changes in regional brain function.

Given the likely heterogeneity in underlying etiology of MCI, particularly EMCI patients in our data, interpretation of group differences or the lack of a difference (CN versus EMCI) is limited with regard to the sensitivity and specificity of detection for prodromal AD. Further, correlation with disease severity is also non-ideal in this analysis as the potential for group effects and relative proportion of the presence of AD could influence findings. Longitudinal data, as well as comparison with molecular measures of AD (e.g. amyloid imaging), will be revealing for determining the predictive value of this modality on an individual case level and longitudinal scanning will give better indication of the sensitivity of this modality to track disease progression. Finally, even with the data cleaning strategies employed, some participants did not have adequate quality ASL data to be included in the analysis. In nearly all cases, we suspect that poor data had been degraded by motion. While not a significant proportion of patients, in the context of a clinical trial, this could be problematic and remediative strategies, such as a second acquisition within each scanning session, could be pursued.

It is important to put the current ASL MRI findings in the context of other biomarker studies and current models of the relationship of these markers with disease stage. One influential conceptualization is that biomarkers of AD can be divided into those that are sensitive to cerebral Aβ-plaque deposition (e.g. amyloid imaging, CSF Aβ) and those that are sensitive to neuronal dysfunction and injury (e.g. FDG PET, structural MRI, CSF total tau/phosphorylated tau) (Albert et al., 2011; Jack et al., 2010, 2013a). While not without some degree of controversy, it has become generally accepted that amyloid-based markers become abnormal first, likely prior to symptom onset, reflecting the antecedent role of amyloid accumulation in the disease course. Both in theory and with some support in the literature, alterations in amyloid are then followed by evidence of neurodegeneration (Buchhave et al., 2012; Jack et al., 2011; Landau et al., 2012). Some work has suggested that the functional changes detected by FDG PET may precede those of atrophy revealed by structural imaging and are potentially more accurate in prediction of dementia from MCI (Landau et al., 2010; Mosconi et al., 2006), but other work has found abnormalities in these measures suggest that they may occur in a more parallel fashion (Bateman et al., 2012). In general, neurodegenerative biomarkers appear more sensitive to disease status in prodromal and symptomatic stages of disease, as amyloid may plateau prior to these stages (Jack et al., 2009, 2013b; Vemuri et al., 2010).

It is likely that ASL MRI will provide similar information to FDG PET. Indeed, as noted above, a recent FDG PET study in a partially overlapping subset of the ADNI data produced comparable findings with regard to the group effects observed here (Landau et al., 2012). However, it is certainly possible that there may be some discordance between CBF and metabolism, as has been reported in the hippocampus (Alsop et al., 2008; Dai et al., 2009), and that these measures may prove differentially sensitive to different disease stages. Some work, including the current findings, support the notion that AD biomarkers are likely to be complementary for determining both disease etiology and stage (Jack et al., 2009; Landau et al., 2012). ADNI offers a unique opportunity to compare the sensitivity of most of the major neuroimaging and biofluid measures, as well as more novel ones, for the diagnosis and disease tracking. Much more work will be needed to determine where ASL MRI will fit into the cascade of biomarker abnormality through the duration of the disease from preclinical to severe dementia. The relative timing, sensitivity, and dynamic range of these various measures will ultimately determine their role in clinical research and practice.

In conclusion, the current data support the sensitivity of ASL MRI to prodromal and early AD CBF alterations in the context of a multi-site study using a commercial ASL sequence. Further, this modality in combination with structural markers of atrophy, which can be obtained within the same scanning session, appeared to predict disease severity in a complementary manner, supporting the notion that multi-modality approaches may be most useful for tracking disease progression.

## Figures and Tables

**Table 1:** Demographic and Neuropsychological Data.

	CN (n = 44)	EMCI (n = 31)	LMCI (n = 30)	AD (n = 15)
Age	73.0 (7.0)	68.7 (7.2)⁎	72.3 (7.4)	75.6 (8.8)
Education	16.3 (2.5)	16.6 (2.8)	16.6 (2.9)	15.7 (2.8)
Female:male	26:18	14:17	14:16	4:11⁎
MMSE	29.0 (1.3)	28.6 (1.4)	27.7 (2.0)⁎⁎	22.4 (1.6)⁎⁎
CDR	0.0 (0.0)	0.5 (0.0)	0.5 (0.0)	0.9 (0.4)
CDR Sum of Boxes	.02 (0.1)	1.0 (0.7)⁎⁎	1.7 (1.0)⁎⁎	4.9 (1.8)
WMS-LM immediate	14.0 (3.3)	11.1 (2.5)⁎⁎	7.6 (2.8) ⁎⁎	3.9 (2.6) ⁎⁎
WMS-LM delayed	13.5 (3.2)	9.0 (1.7)⁎⁎	4.2 (2.8)⁎⁎	1.3 (2.2)⁎⁎
AVLT sum of trials 1–5	44.1 (10.4)	44.1 (11.9)	33.9 (11.4)⁎⁎	21.2 (8.9)⁎⁎
AVLT 5-min delayed recall	8.1 (3.8)	8.7 (4.5)	5.1 (3.8)⁎⁎	1.4 (1.5)⁎⁎
AVLT 30-min delayed recall	6.9 (4.2)	7.4 (5.0)	3.8 (3.8)⁎⁎	0.6 (1.2)⁎⁎
Trails A (s)	32.6 (11.1)	29.4 (7.7)	41.7 (18.8)⁎	55.9 (28.8)⁎⁎
Trails B (s)	87.8 (51.8)	73.7 (31.9)	133.8 (86.7)⁎⁎	181.6 (68.0)⁎⁎
BNT total	28.1 (2.3)	27.5 (2.6)	25.9 (3.4)⁎⁎	23.3 (4.9)⁎⁎
Category fluency (animals)	22.4 (6.5)	20.3 (4.1)	16.9 (5.7)⁎⁎	12.1 (4.9)⁎⁎

Note: Standard deviations are in parentheses. Abbreviations: CN: cognitive normal subjects; EMCI: early mild cognitive impairment patients; LMCI: late MCI patients; AD: Alzheimer's Disease; MMSE: Mini–Mental State Examination; CDR- Clinical Dementia Rating; WMS-LM: Wechsler Memory Scale–Revised Logical Memory II; AVLT: Auditory Verbal Learning Test; BNT: Boston Naming Test.

⁎ p < 0.05 compared to the control group.

⁎⁎ p < 0.01 compared to the control group.

## Appendix A Supplementary data

Supplementary Table 1. Number of subjects included and excluded for each site and each group. The number series delimited by "," in each table cell mean the number for health control, early MCI, late MCI, and AD, respectively from left to right. Supplementary Figure 1. ASL perfusion MRI data processing pipeline. Supplementary Figure 2. A flow chart for the adaptive outlier cleaning process.
