# Supplementary material for: Arterial spin labeled MRI in prodromal Alzheimer's disease: A multi-site study
Source: Neuroimage Clin. 2013 Apr 30;2:630–6. doi: 10.1016/j.nicl.2013.04.014 (PMC3777751; doi:10.1016/j.nicl.2013.04.014)
Supplement: Supplementary file 1 — Supplementary Table 1. Number of subjects included and excluded for each site and each group. The number series delimited by "," in each table cell mean the number for health control, early MCI, late MCI, and AD, respectively from left to right. Supplementary Figure 1. ASL perfusion MRI data processing pipeline. Supplementary Figure 2. A flow chart for the adaptive outlier cleaning process. [file mmc1.docx]

Supplementary Table 1. Number of subjects included and excluded for each site and each group. The number series delimited by “,” in each table cell mean the number for health control, early MCI, late MCI, and AD, respectively from left to right.

| SITE ID | # of subjects | |
| --- | --- | --- |
|  | included | excluded |
| 9 | 1,0,2,0 | 0,0,0,0 |
| 11 | 4,2,1,0 |  |
| 14 | 5,1,2,2 |  |
| 23 | 3,1,6,1 |  |
| 24 | 1,1,1,2 |  |
| 36 | 1,0,0,0 | 1,0,1,0 |
| 37 | 3,1,5,1 | 1,0,0,0 |
| 67 | 0,2,0,0 | 0,0,1,0 |
| 73 | 4,7,3,0 |  |
| 116 | 5,3,1,5 |  |
| 123 | 1,1,2,1 |  |
| 128 | 3,3,1,0 | 0,0,1,0 |
| 135 | 3,3,2,2 |  |
| 141 | 1,0,0,0 | 0,1,1,0 |
| 153 | 4,3,2,1 | 1,0,0,0 |
| 941 | 6,2,2,0 | 1,0,0,0 |

**
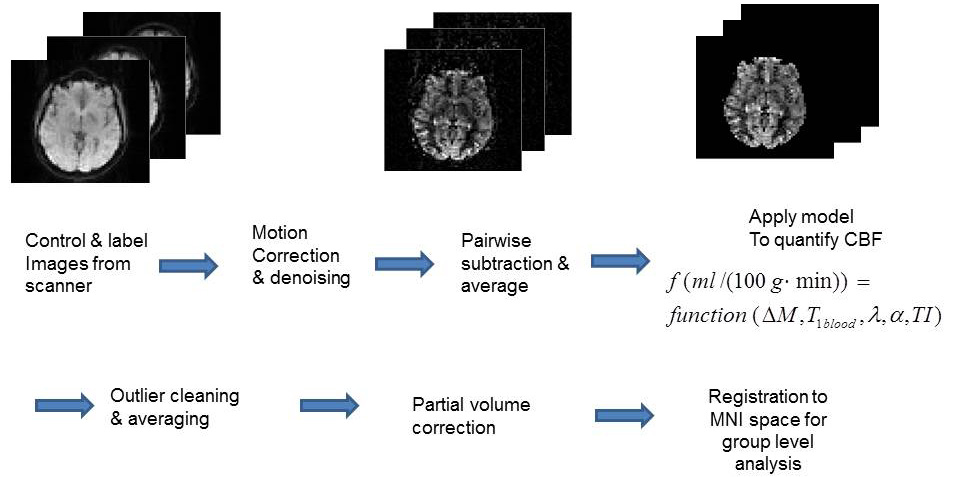
**

Supplementary Figure 1. ASL perfusion MRI data processing pipeline.


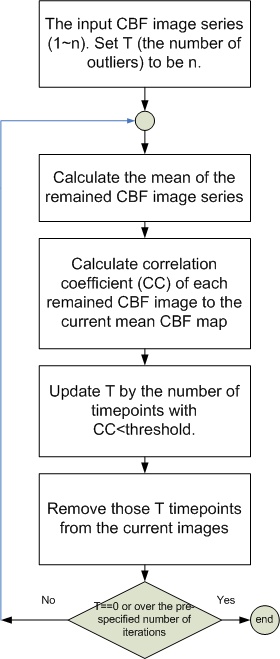


Supplementary Figure 2. A flow chart for the adaptive outlier cleaning process.


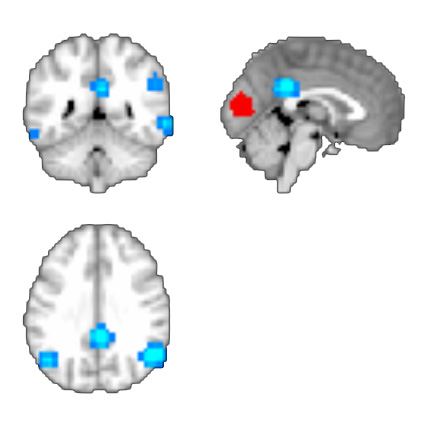


Supplementary Figure 3. Illustration of the meta-ROI (blue color) and the control ROI (in red) used in the main text.
